# EFFECT OF D-ALLULOSE ON INSULIN RESISTANCE AND ENDURANCE ABILITY: IS D-ALLULOSE AN EXERCISE MIMETIC?

**DOI:** 10.1093/geroni/igae098.2833

**Published:** 2024-12-31

**Authors:** Teruhiko Koike, Yang Gou, Bingyang Liu, Yukie Natsume, Takamasa Tsuzuki, Kamal Shahriar, Ryoichi Banno

**Affiliations:** Nagoya University, Nagoya, Aichi, Japan; University of Washington, Seattle, Washington, United States; Zhejiang University, Hangzhou, Zhejiang, China (People’s Republic); Nagoya university of economics, Inuyama-shi, Aichi, Japan; Meijo University, Nagoya, Aichi, Japan; Nagoya University Graduate School of Medicine, Nagoya, Aichi, Japan; Nagoya University, Nagoya, Aichi, Japan

## Abstract

D-Allulose, a rare sugar and C3-epimer of D-fructose has been proposed as a candidate for dietary restriction mimetics via inhibition of glycolysis. Anti-hyperglycemic and anti-obesity effects have been reported in rodents and human subjects. Here, we report that D-allulose increases the glucose infusion rate in the two-step hyperinsulinemic-euglycemic clamp test in high-fat or high-sucrose diet-fed rat models. Consistently, the insulin-induced phosphorylation of AKT in the skeletal muscle was higher with D-allulose administration with decreased levels of inflammatory cytokine, TNF-α. We also report the effect of D-allulose on insulin sensitivity and aerobic performance in a healthy mice model and compare them with exercise training effects. D-allulose enhanced insulin sensitivity, increased running distance, and maximal aerobic speed (MAS). The improved exercise performance was associated with lower blood lactate levels and increased liver glycogen contents. D-allulose also enhanced the phosphorylation of AMP-activated protein kinase (AMPK) and acetyl-CoA carboxylase (ACC), and the expression of peroxisome proliferator-activated receptor γ coactivator 1α (PGC-1α). Our results indicate that D-allulose administration improves insulin sensitivity and enhances endurance ability like exercise training.

